# Honey Bees (*Apis mellifera* Hymenoptera: Apidae) Preferentially Avoid Sugar Solutions Supplemented with Field-Relevant Concentrations of Hydrogen Peroxide Despite High Tolerance Limits

**DOI:** 10.1093/jisesa/ieab102

**Published:** 2021-12-27

**Authors:** Lewis J Bartlett, Carlos Martinez-Mejia, Keith S Delaplane

**Affiliations:** 1 Center for the Ecology of Infectious Diseases, Odum School of Ecology, University of Georgia, Athens, GA 30602, USA; 2 New York University, New York, NY 10003, USA; 3 Department of Entomology, University of Georgia, Athens, GA 30602, USA

**Keywords:** hydrogen peroxide, social immunity, foraging behavior, feeding choice, toxicity

## Abstract

Honey bees (*Apis mellifera* L. Hymeoptera: Apidae) use hydrogen peroxide (synthesized by excreted glucose oxidase) as an important component of social immunity. However, both tolerance of hydrogen peroxide and the production of glucose oxidase in honey is costly. Hydrogen peroxide may also be encountered by honey bees at high concentrations in nectar while foraging, however despite its presence both in their foraged and stored foods, it is unclear if and how bees monitor concentrations of, and their behavioral responses to, hydrogen peroxide. The costs of glucose oxidase production and the presence of hydrogen peroxide in both nectar and honey suggest hypotheses that honey bees preferentially forage on hydrogen peroxide supplemented feed syrups at certain concentrations, and avoid feed syrups supplemented with hydrogen peroxide at concentrations above some tolerance threshold. We test these hypotheses and find that, counter to expectation, honey bees avoid glucose solutions supplemented with field-relevant hydrogen peroxide concentrations and either avoid or don’t differentiate supplemented sucrose solutions when given choice assays. This is despite honey bees showing high tolerance for hydrogen peroxide in feed solutions, with no elevated mortality until concentrations of hydrogen peroxide exceed 1% (v/v) in solution, with survival apparent even at concentrations up to 10%. The behavioral interaction of honey bees with hydrogen peroxide during both within-colony synthesis in honey and when foraging on nectar therefore likely relies on interactions with other indicator molecules, and maybe constrained evolutionarily in its plasticity, representing a constitutive immune mechanism.

Eusocial living is phylogenetically rare, having evolved only around 24 times (Bourke 2011). One of its constraints is thought to be parasite pressure as a consequence of high host densities and contact rates, high individual relatedness, stable nest environments, and vulnerable food stores (Schmid-Hempel 1998). All these conditions are met in honey bees (*Apis mellifera* L. Hymeoptera: Apidae), who demonstrate highly derived eusociality and associated challenges in defending against parasites (Brosi et al. 2017). Group living seems to have counterintuitively led to the attrition of individual immune genes (Evans et al. 2006), instead favoring investment in social mechanisms such as propolis gathering and social medication (Evans and Spivak 2010, Borba et al. 2015, Spivak et al. 2019). For example, honeybees are known to forage on specific plants to obtain necessary antimicrobials (Erler and Moritz 2016, Richardson et al. 2016, Tihelka 2018, Bernklau et al. 2019), reduce investment in personal immunity in favor of colony-level defense (Borba and Spivak 2017), and tailor food store choices in response to parasitic pressures (Gherman et al. 2014). Through these mechanisms and others, honeybees outsource individual immunity to group-level social immunity. Much of this is emphasized in brood rearing, as honeybee larvae are vulnerable to infectious or parasitic agents (Brosi et al. 2017), with no consistent core gut microbiome (Martinson et al. 2012) and a reliance on worker nurses for total care, leading to dependence on colony-level parasite defenses.

This emphasis on social, community-level defenses is evolutionarily interesting, but also critical to the applied ecological topic of managing honeybee health. Manged honeybees are essential to many agricultural systems, especially in the United States of America (U.S.), as a pollination service (Calderone 2012, Rader et al. 2012) and are further recognized for their outright economic and cultural value (Bingham 2006, Watson et al. 2011, Mace et al. 2012). However, honeybee populations in the U.S. have suffered declines and growth suppression due to interacting ecological factors (Aizen and Harder 2009, Potts et al. 2010, vanEngelsdorp and Meixner 2010), leading to loss of livelihoods and unmet demand for pollination services (Ferrier et al. 2018). Consequently, there is an applied need to better understand how honey bees defend themselves against pathogens and parasites as well as a fundamental scientific interest in their social-immunity investment.

One mechanism of colony-level immunity to parasites is the production of hydrogen peroxide (H_2_O_2_) in honeybee food stores (López-Uribe et al. 2017). Honeybees store food to survive dearths (notably, winter), and to feed the growing brood. Stored food is vulnerable to parasitism by both micro- and macro-parasites; microbial growth in stored honey is inhibited principally due to its low water content, however, H_2_O_2_ synthesized from excreted glucose oxidase (GOX) also putatively assists in deterring both of these (White et al. 1963, Brudzynski et al. 2011, Bucekova et al. 2014). However, producing GOX and evolving tolerance of H_2_O_2_ represent costly investments in social immunity (Ohashi et al. 1999, Seehuus et al. 2006, Korayem et al. 2012, López-Uribe et al. 2017, Jones et al. 2018); these costs are broadly unexplored, both in the context of fundamental colony function and applied management. There is evidence that floral nectar sources vary in their adequacy as substrates for GOX H_2_O_2_ synthesis, leading to honey with a range of H_2_O_2_ concentrations, as reviewed by Brudzynski (2020). While the coarse biochemistry of GOX activity in honey is increasingly well understood (Brudzynski 2020), there is little understanding of how honey bees regulate or monitor this costly investment in H_2_O_2_ production, particularly as H_2_O_2_ reaches some equilibrium in honey due to both its inherent instability and its ready reaction with glucose to form gluconic acid (Mao 2017), which may act as an indicator of H_2_O_2_ content.

Tolerance of H_2_O_2_ in honey is costly in honey bees (Korayem et al. 2012), and further to this Jones et al. (2018) showed that the production of H_2_O_2_ via GOX by honey bees is also costly. However, López-Uribe et al. (2017) documented that there was no up-regulation in GOX production/activity in response to increased pathogen pressure, a result which seems maladaptive and perhaps signals significant evolutionary constraints on the ability of honey bees to fine-tune this aspect of colony immune function. This suggests that H_2_O_2_ tolerance and production are constitutive immunity costs, which may be critical to understanding honey bee immunological evolution as constitutive trade-offs in immunity often carry significant costs to balance their benefits (Cressler et al. 2015, Bartlett et al. 2018, Jones et al. 2018, van Alphen and Fernhout 2020).

Given that GOX is costly to produce and H_2_O_2_ is an important component in honey, we would expect honey bees to show a preference when foraging or feeding on H_2_O_2_-supplemented diets—up to some limit above which H_2_O_2_ becomes too toxic or costly to consume. H_2_O_2_ is present in some plant nectars (Alvarez-Pérez et al. 2012, Nocentini et al. 2015), however, this is a little-investigated part of bee foraging behavior (Schmitt et al. 2021). We explore the behavioral basis of how H_2_O_2_ influences feeding choice in honey bees to begin building a better understanding of the behavioral capacity of honey bees to regulate this important aspect of colony immune function, with a view to better informing our wider understanding of honey bee health. We test two linked hypotheses: 1) honey bees will preferentially feed on sugar solutions supplemented with hydrogen peroxide at concentrations similar to those encountered in honey or nectar; and 2) honey bees will avoid hydrogen peroxide supplemented sugar solutions when the concentration of hydrogen peroxide approaches their tolerance limit as found through a dose–response analysis.

## Methods

### Experimental Design and Honey Bee Collection

We ran six main assays; assays were not run concurrently due to both labor and incubator space limits. We ran two mortality assays, both used six concentrations of H_2_O_2_ in sucrose solution: one with concentrations at 0, 100, 500, 1,000, 5,000, 10,000 µg/ml and one assay at 0, 2, 4, 6, 8, 10% (v/v—approximately 0 – 100,000 µg/ml) H_2_O_2_. We collected bees from three colonies, with two technical-replicate assay cages per dose per colony (36 cages total for each mortality assay). Bees for mortality assays were collected by shaking adult bees from brood frames into an aluminum tray and scooping approximately 80 bees directly into mortality cages in the field. We ran four choice assays, two at 50 µg/ml supplemented H_2_O_2_ and two across a range of supplemented H_2_O_2_ concentrations (0.01, 0.1, 1, 10, 100, 1,000 µg/ml H_2_O_2_), using either glucose or sucrose solutions. We focused sampling much more densely on the 50 µg/ml H_2_O_2_ concentration as this reflected values commonly found in honey; for example, H_2_O_2_ concentrations for wild-foraged honey in the literature (converted here from µg/g to µg/ml at 1 ml honey = 1.4 g) show ranges of 35–56 µg/ml in Strelec et al. (2018), 26–58 µg/ml in Pasias et al. (2018), 17–45 µg/ml in Farkasovska et al. (2019), and 0.84–56 µg/ml in Cebrero et al. (2020). In all choice experiments, we collected approximately 12–15 bees into assay cages directly from brood frames by ‘rolling’ bees from the frame surface. For the 50 µg/ml H_2_O_2_ assays, we had 24 replicate assay cages taken from either three (sucrose assay) or four (glucose assay) colonies. For the choice assays across concentrations, we used six different concentrations and six replicate assay cages at each concentration reflecting either six (sucrose) or three (glucose) donor colonies. Donor colonies were not necessarily shared across any assays, most assays were sampled from entirely different colonies at different apiaries to avoid exposure to external ongoing field experiments.

### Choice and Mortality Assays

Assays used typical honey bee toxicology cages, which were modified from food-grade plastic with either one or two openings to insert sugar solution feeders. Feeders were modified veterinary grade luer-slip syringes (ThermoFisher USA). Mortality assay cages used a single 3 ml feeder filled with exactly 3 ml of test solution suspended from the top of the cage. Choice assay cages used two 1 ml feeders (one control solution, one test solution) both suspended from the top of the cage. All assay cages were placed immediately in a dark incubator at 30°C and 70% relative humidity. Choice assays ran for 24 hr, after which the remaining amount of sugar solution in each feeder syringe was read directly from the syringe measure. Mortality assays ran for 48 hr; upon removal from the incubator, any cages with no remaining sugar solution were flagged for starvation. The number of dead, immobile, and/or alive bees were counted; cohorts were then euthanized by freezing and the total number of bees was counted exactly by hand.

### Test Solutions

All sugar solutions provided were 33% sugars by mass (a ‘1:2 by weight’ sugar feed in beekeeping terminology). For each experiment, a single stock solution was made up at a 1:1 mass ratio (50% sugars w/w) and then subsequently diluted down for each test solution using water and/or hydrogen peroxide solution in the necessary ratios. We used both laboratory grade sucrose (ThermoFisher, USA) and glucose (ThermoFisher, USA) and dissolved sugars into solution by low heating and continuous stirring; all solutions were made using potable tap water, and the process was designed to reflect the common beekeeper practice of supplementary feeding with sucrose solution in-field. For the high-concentration mortality assay, we used 30% w/w H_2_O_2_ solution (VWR, USA), for all other assays we used 3% w/w H_2_O_2_ solution (VWR, USA). All solutions were freshly prepared at the start of each assay.

We tested excess experimental solutions from each assay at the beginning of each experiment to confirm H_2_O_2_ concentrations using commercial environmental water ecotoxicological monitoring kits (CHEMetrics, USA—Product K5510), which are a visual colorimetric assay. Where necessary, we serially diluted our solutions to be below the upper detection limit of the monitoring kits. In all cases, solutions at the time of setting up assays were measured to have the expected H_2_O_2_ concentrations. We also tested remaining test solution H_2_O_2_ concentrations at the 24 (or 48 where appropriate) hr marks at the end of each assay. In the case of the sucrose solutions, H_2_O_2_ concentrations appeared stable and were measured to have the same H_2_O_2_ concentrations at the start and end of the assays. For glucose solutions, H_2_O_2_ rapidly depleted due to reacting with the glucose, and consistently showed little detectable residual H_2_O_2_ at the end of the assays. This was expected given the documented chemistry of saccharide-H_2_O_2_ reactions, where sucrose is comparatively unreactive with H_2_O_2_ whereas glucose is known to more readily react in solution with H_2_O_2_ to form gluconic acid. We did not monitor for microbial growth, as these experiments were initially designed to emulate the practice of sugar feeding as used by practitioners (beekeepers).

### Statistical Analysis

All analysis was undertaken in the statistical programming language R v3.6.1 (R Core Team 2019). We used a generalized linear mixed modeling (GLMM) framework for the majority of the analysis, using the ‘afex’ package (Singmann et al. 2019) (which wraps around the ‘lme4’ package (Bates et al. 2015)) to construct models and test for significance using type-III ANOVAs, with subsequent estimation and comparison of effects sizes using the ‘emmeans’ package (Lenth 2019). For the two mortality data sets, we used binomial GLMMs with a response variable of whether a bee survived or died, hierarchical random effects of colony and cage (nested under colony), and fixed predictors of supplemented H_2_O_2_ concentration (continuous numeric) and a binary true/false ‘starvation’ predictor. For the choice assay data sets, we first analyzed each sugar solution on its own, and then proceeded to a combined analysis for better direct comparison. In the case of the 50 µg/ml assay, each sugar solution was analyzed as a simple one-sample two-way *t*-test with mu = 0 (the null hypothesis: no preference) where the response variable was the offset difference in consumption between control and test solutions. We then combined this data to use a Gaussian-distributed GLMM with ‘Sugar’ (two-factor sucrose vs glucose) as a fixed predictor and a random effect of colony nested under sugar type, also testing for significance of the intercept. For choice assay data spanning multiple concentrations, we first analyzed each sugar separately with offset difference in consumption between control and test solutions as the response variable, the concentration of supplemented H_2_O_2_ as a continuous fixed predictor, and the colony as a random effect, also testing for significance of the intercept. When we combined these data sets, we used the same framework but with interacting fixed predictors of sugar type and concentration, and colony nested under sugar type as the random effect. All data and analysis are made available via a Zenodo-archived GitHub repository release (https://doi.org/10.5281/zenodo.5706542).

## Results

**Fig. 1.**
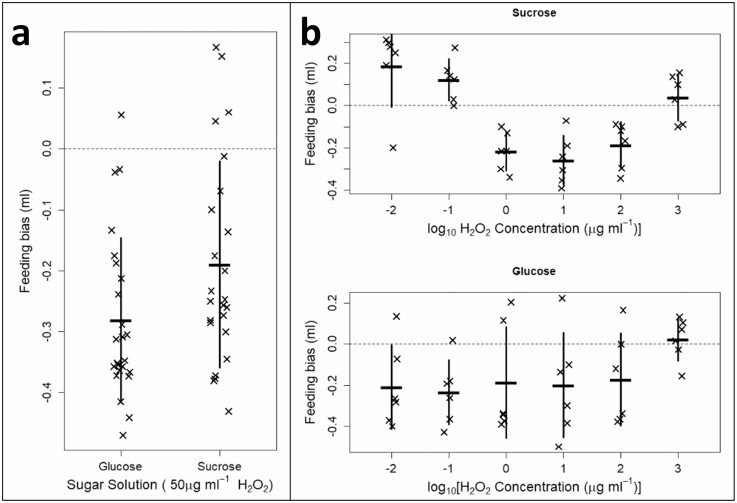
Choice assay data for both sucrose and glucose solutions, whereby each point is one feeding cage. Response variable in all panels is an offset feeding bias, where a value of ‘0’ represents indiscriminate feeding between control and H_2_O_2_-supplemented test sugar solutions, a positive value indicates a preference for feeding from test solutions, and a negative value indicates a preference for the control solution (i.e., an avoidance of the test solution). Panel **a** compares the densely sampled choice assays at 50 µg/ml H_2_O_2_, corresponding approximately to values found in honey. Panel **b** compared across six orders of magnitude of H_2_O_2_ concentrations, spanning 0.01–1,000 µg/ml supplemented H_2_O_2_.

We found evidence that honey bees avoided solutions supplemented with H_2_O_2_. Firstly when independently examining both the 50 µg/ml H_2_O_2_ glucose solution (Fig. 1a, *t*_23_ = −10.19, *p* < 0.001), and sucrose solution (Fig. 1a, *t*_23_ = −5.49, *p* < 0.001) choice assays, honey bees preferentially consumed from the control sugar solutions in both cases. This remained true when, at these 50 µg/ml concentrations, a grouped analysis showed avoidance of the test solutions (*F*_1,4.84_ = 56.94, *p* < 0.01); however there was no difference in the strength of avoidance of the test solutions when comparing glucose to sucrose assays (*F*_1,4.84_ = 2.83, *p* = 0.206); on average 21.9% (95% C.I.: 11.3–32.4%) of the consumed solution was test solution when using glucose, and 31.0% (95% C.I.: 18.6–44.4%) when using sucrose.

Somewhat consistent with this more densely sampled single-concentration data set, there was some evidence of avoidance of test solutions across a range of concentrations (Fig. 1b), however, these findings were not clear cut. There was no strong evidence of avoidance (*F*_1,7.80_ = 3.06, *p* = 0.120) or effect of concentration (*F*_1,29_ = 0.94, *p* = 0.340) when looking solely at the sucrose solution data. Examining the glucose data, again no significant overall avoidance was observed (*F*_1,2.10_ = 5.59, *p* = 0.136) however an effect of concentration was found where glucose test solutions supplemented with higher concentration H_2_O_2_ were avoided less (*F*_1,32_ = 10.18, *p* = 0.003). When analyzed as a grouped data set, a significant tendency towards avoiding test solutions was however observed (*F*_1,7.17_ = 12.82, *p* = 0.009), although this did not differ between sugar solution types (*F*_1,7.17_ = 3.17, *p* = 0.117), and an effect of concentration where avoidance was strongest at the lowest concentrations of supplemented H_2_O_2_ was observed (*F*_1,61.11_ = 7.12, *p* = 0.010) but was again not found to differ between sugar solutions (*F*_1,61.11_ = 1.28, *p* = 0.262).

**Fig. 2.**
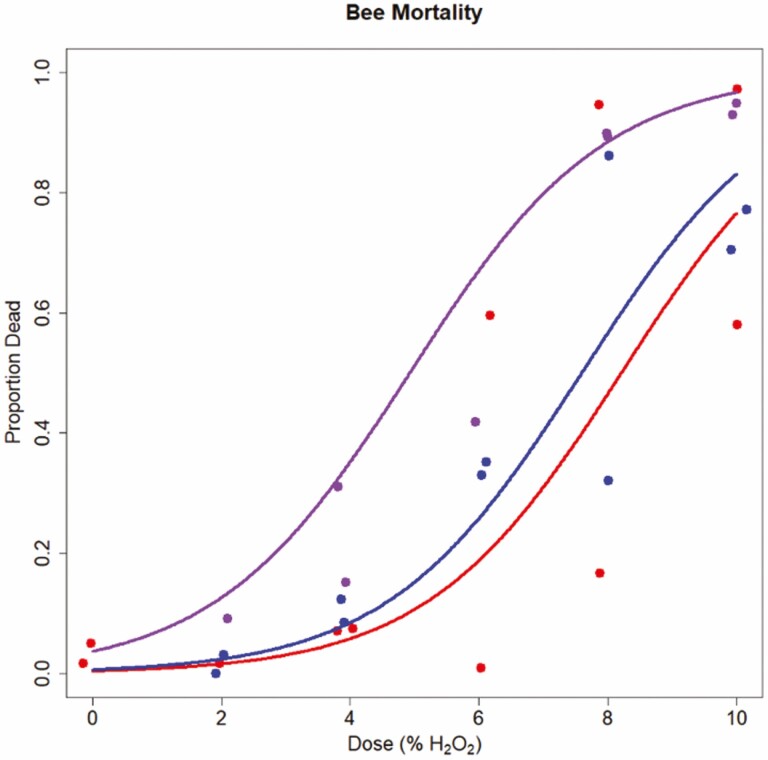
Dose mortality curves for the higher-dose mortality assay undertaken. Each point corresponds to a single exposure cage, with plot colors corresponding to each of the three colonies samples were taken from, with two cages per colony per dose. Cages which were identified as having suffered from starvation are excluded from plotting for clarity (but are included in statistical analyses). X-axis uses a volumetric concentration, corresponding to up to approximately 100,000 µg/ml H_2_O_2_.

In analyzing the mortality assays, we found no clear evidence of supplemented H_2_O_2_ toxicity at the lower concentrations we examined (between 0 and 10,000 µg/ml H_2_O_2_, χ ^2^_1,4_ = 3.40, *p* = 0.065; see Supp Fig. 1 [online only]). We did find evidence for toxicity at higher concentrations (between 0 and 10% v/v, corresponding to approximately up to 100,000 µg/ml H_2_O_2_), see Fig. 2 (χ ^2^_1,5_ = 30.04, *p* < 0.001) where we successfully fit dose–response curves. However, even at the highest concentrations we still failed to detect consistent complete mortality in all replicates, with substantial survivorship (Fig. 2).

## Discussion

Honey bees avoided feed solutions supplemented with H_2_O_2_, despite their investment in and use of H_2_O_2_ in honey; this directly refutes our first hypothesis. This was most clear at concentrations meant to emulate those found naturally in honey. The clarity of this avoidance behavior may be muddled when we look across concentrations (Fig. 1), but we still clearly show that our expectation that honey bees preferentially forage on the H_2_O_2_-supplemented test solutions is incorrect.

For the sucrose solution choice assays, hydrogen peroxide concentrations remained reasonably stable throughout the experiments as confirmed by serial dilutions and visual colorimetric assays (sucrose in solution is fairly stable with increasing H_2_O_2_ concentration, see Sartori et al. (2017)). At a minimum, honey bees are either incapable of directly detecting hydrogen peroxide in sucrose solution at these concentrations (if we accept the conservative hypothesis that there was no substantial avoidance behavior in the sucrose assays) or are actively avoiding it either through direct detection or aversive postfeeding reactions (e.g., nausea) causing them to learn which feeder to avoid. There is, to our knowledge, no good evidence of any animal being able to directly detect hydrogen peroxide through chemoreceptors; this is expected given the small, unstable nature of the molecule and its metabolic ubiquity. We tentatively, therefore, offer evidence that this is a ‘learned response’ mediated by postfeeding physiological signaling. This would suggest that the role of H_2_O_2_ in influencing foraging of pollinators on different nectar sources (Schmitt et al. 2021) is principally indirect via changing nectar microbiomes and olfactory cues via interaction with other aspects of nectar chemistry; it is possible that microbial cues also played a part in our experiments, although we did not investigate this formally.

For the glucose solution choice assays, there was stronger evidence of avoidance of the supplemented test solutions (Fig. 1). Our postassay testing of the test solutions demonstrated that, unlike the sucrose assays, hydrogen peroxide rapidly depleted due to reacting with the glucose (as expected from well-characterized saccharide-peroxide chemistry (Mao 2017)). This reaction principally generates gluconic acid, forming a gluconate ion in solution, which is readily sensed via chemoreceptors by many animals (Goldberg and Rokem 2009). Gluconate is naturally found in honey (Karaffa and Kubicek 2021) due to the same reaction of hydrogen peroxide with glucose; our glucose-based test solutions are chemically much more similar to honey than our sucrose solutions (which better emulate nectar). However, we see strong evidence of an avoidance effect in the glucose solutions despite little active H_2_O_2_ and the assumed presence of this gluconate ion; we observe honey bees avoiding the gluconate solution despite its presence in honey and its potential role as an indicator of hydrogen peroxide activity. The lack of other stimuli in the solution, for example, the lactone of gluconate (glucono delta-lactone ‘GDL’) which is present in honey from the generation of hydrogen peroxide via glucose oxidase activity, may partially account for the lack of preferential foraging on these test solutions. It may also be indicative of a true avoidance of H_2_O_2_ indicators found in honey. Finally, it is possible that much more nuanced decision-making is being made by the honey bees, where high is consumed only as a ‘last option’ *because* it is a high-investment stored food, and so better serves long-term storage, use in brood feeding, or self-medication purposes. Future work may characterize whether honey bees preferentially forage on sugar solutions supplemented with either gluconic acid, GDL, or both in combination, and whether this depends on the age of the honey bees (which determines their task allocation). Gluconate is not known to be toxic to honey bees, lending us no speculative explanation for the avoidance behavior we observe, except as an indicator of the costs of oxidative stress mitigation when H_2_O_2_ is consumed.

Our initial hypothesis speculated that honey bees would preferentially forage on H_2_O_2_-supplemented solutions due to its antimicrobial effects, presence in honey and nectars, and the cost of investment in glucose oxidase to generate H_2_O_2_ in honey. However counter to this, and in better conceptual agreement with our results, it is also costly to mitigate the oxidative stress caused by high hydrogen peroxide concentrations encountered when feeding (Korayem et al. 2012). This cost of oxidative stress mitigation could account for the avoidance behaviors we observe; speculatively, honey bees may use gluconate as an indicator of the potential oxidative stress imposed by the honey they are consuming. However, this hypothesis is weakened by the remarkable robustness we observe in honey bees when trying to characterize the oral toxicity of hydrogen peroxide in *A. mellifera*. We saw no indication of mortality caused directly by H_2_O_2_ or via starvation due to feeding inhibition at or below 1% H_2_O_2_ (~10,000 ug/ml) H_2_O_2_ (Fig. 2, Supp Fig. 1 [online only]), at least one order of magnitude above concentrations encountered in either honey (Brudzynski 2020) and significantly higher than that seen in nectar (Schmitt et al. 2021). While our simple mortality assays do not capture the sublethal costs of exposure to high H_2_O_2_ concentrations in feed solutions, they do highlight the capability of adult honey bees to survive and avoid starvation when given no choice but to consume high-concentration H_2_O_2_ solutions. The mismatch in the potential avoidance of H_2_O_2_ concentrations in sucrose solution (Fig. 1) and when we detect mortality associated with H_2_O_2_ concentration (Fig. 2) is quite extreme—around three orders of magnitude if comparing midpoints across the assays. If protection from H_2_O_2_-caused oxidative stress is constitutively expressed by adult honey bees at some base rate then there is little obvious benefit in avoiding these low-level concentrations; rather, these results may provide circumstantial evidence of the facultative ability of honey bees to quickly upregulate their (costly) oxidative protection measures; putatively, this has been linked to coping with metabolic oxidative stress during flight (Margotta et al. 2018) and worker repurposing of reproductive pathways (Seehuus et al. 2006). This would represent a ‘behavioral defense first, physiological defense second’ series of mechanisms of protection from orally administered H_2_O_2_ oxidative stress.

There are many possible experiments one could design to explore the tolerance, preference, or avoidance of H_2_O_2_ (or its downstream reaction products in glucose solution) by adult honey bees. For example, our mortality data (Fig. 2, Supp Fig. 1 [online only]) is unsuitable for examining if honey bees consume less sucrose solution at H_2_O_2_ concentrations avoided in the choice experiments as feeding was not ad-libitum, starvation occurred, and there is an obvious confound of bee mortality at high doses; other experiments would be required to show if consumption is reduced when the only ‘option’ is high-H_2_O_2_ sugar solutions). It is overall clear from our results that the mechanisms underpinning honey bee behavioral interactions with H_2_O_2_ are likely complex, context dependent, and rely on the detection of specific indicator molecules in honey. It remains unknown if and how honey bees can quantify H_2_O_2_ concentration in sugar solutions, and how this feedback influences their investment in excreted glucose oxidase during honey production, or their feeding choices when foraging, feeding themselves, provisioning brood, or self-medicating. López-Uribe et al. (2017) found no evidence for the up-regulation of glucose oxidase production when honey bees were challenged with a pathogen, suggesting that honey bee control over H_2_O_2_ concentrations in their honey is less fine-tuned and principally environmentally dependent. This is plausibly in agreement with the extremely wide-ranging concentrations of H_2_O_2_ observed in honey from different locations and botanical sources (Brudzynski 2020). Rather, it may be that honey bees follow simple rules for generating H_2_O_2_ in their stored food products, leading to highly variable H_2_O_2_ concentrations and subsequent facultative up- or down-regulation of their antioxidative protective pathways—a line of reasoning our assays agree with when comparing the concentrations at which honeybees avoid H_2_O_2_-supplemented solutions and at which they can no longer tolerate increasing H_2_O_2_, as well as the mixed evidence for avoidance we observe. However, their avoidance of sucrose solutions fortified with H_2_O_2_ concentrations is well within the range of what is observed in nectar (Schmitt et al. 2021) and maybe a broadly under-studied aspect of foraging choices made by honey bees in the landscape.

## Supplementary Material

ieab102_suppl_Supplementary_Material
