# Supplementary material for: Honey Bees (Apis mellifera Hymenoptera: Apidae) Preferentially Avoid Sugar Solutions Supplemented with Field-Relevant Concentrations of Hydrogen Peroxide Despite High Tolerance Limits
Source: J Insect Sci. 2021 Dec 27;22(1):2. doi: 10.1093/jisesa/ieab102 (PMC8711758; doi:10.1093/jisesa/ieab102)
Supplement: ieab102_suppl_Supplementary_Material [file ieab102_suppl_supplementary_material.pdf]

Supplementary Material

Honey bees (Apis mellifera Hymenoptera: Apidae) preferentially avoid sugar solutions supplemented with field-relevant concentrations of hydrogen peroxide despite high tolerance limits.

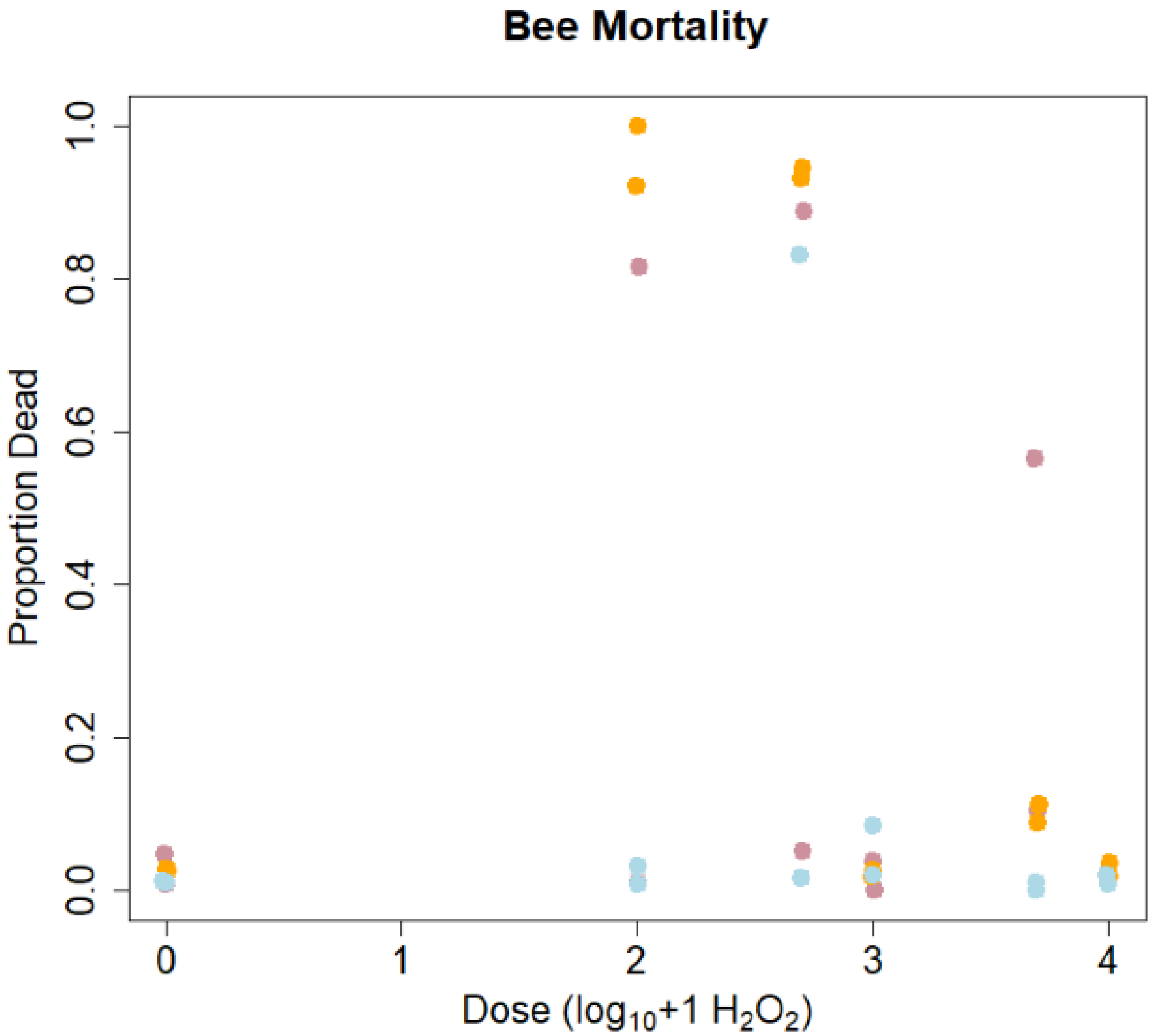

Figure S1 – Raw data, including starved cages, for the lower-concentration mortality experiment seeking to establish a dose-response for hydrogen peroxide. No significant effect of dose was found on mortality at these lower doses (corresponding to 0 – 10,000 ugml^{-1} H_2O_2, or up to approximately 1%). Colours represent different origin colonies (three colonies each donated two cages of bees to each dose).
